# Editorial: Nature and determinants of socio-moral development: theories, methods and applications

**DOI:** 10.3389/fpsyg.2023.1296472

**Published:** 2023-10-20

**Authors:** Alessandra Geraci, Laura Franchin, Aner Govrin, Paola Rigo

**Affiliations:** ^1^Department of Social Science and Education, University for Foreigners, Reggio Calabria, Italy; ^2^Department of Psychology and Cognitive Science, University of Trento, Rovereto, Italy; ^3^Department of Hermeneutics and Cultural Studies, Bar-Ilan University, Ramat Gan, Israel; ^4^Department of Developmental Psychology and Socialization, University of Padua, Padua, Italy

**Keywords:** determinants, socio-moral development, prosociality, child development, infancy

## Introduction

In the last two decades, several developmental researchers explored the ontogenesis of morality, revealing an earlier origin of the moral sense (Hamlin et al., 2007; Hamlin, 2013a), and sophisticated abilities starting from 3 months of age (for a recent review see Woo et al., 2022). In particular, preverbal infants and toddlers demonstrated the ability to make social evaluations of different prosocial actions, such as helping behaviors (e.g., Hamlin et al., 2007, 2010; Surian and Franchin, 2017a; Geraci and Franchin, 2021), distributive actions (e.g., Geraci and Surian, 2011, 2023a; Surian and Franchin, 2017b; Franchin et al., 2019), protecting behaviors (e.g., Kanakogi et al., 2013), affiliative behaviors (e.g., Geraci et al., 2022a) and comforting behaviors (Geraci et al., 2021). Other studies provide evidence for infants' ability to evaluate others' actions by considering the agents' intentions in different prosocial interactions (Hamlin, 2013b; Strid and Meristo, 2020; Geraci et al., 2022b; Geraci and Surian, 2023b). Moreover, infants showed a tendency to punish harming agents (Kanakogi et al., 2022), as well as they expect a bystander to punish antisocial agents (e.g., Hamlin et al., 2011; Kanakogi et al., 2017; Geraci and Surian, 2023b).

To account for this evidence, different explanations for moral development coexist, and yet, there is no consensus in the scientific community, as in the case of the debate between nativist (e.g., Wynn et al., 2018; Ting et al., 2019) and constructivists (Dahl, 2018; Smetana et al., 2018). Moreover, there is little evidence of the impact of the determinants on moral development, such as socio-emotional development and early environmental effects (Govrin, 2014). To broaden the knowledge of socio-moral development, this Research Topic of contributions included articles from different theoretical positions that focus on the methods used and on the applicative aspects. This Research Topic provided a collection of recent advances and novel contributions, empirical studies and review, on the emergence and development of moral sense.

## Overview of contributions

Studies published in this Research Topic addressed two characteristics: a focus on exploring methods that are used by researcher according to different theoretical approaches, and a novel contribution to the literature on the socio-moral development.

On the emergence of morality in the first years of life, the following empirical study shed light on an early-emerging moral sense that works in different domains, and forms expectations on others' behaviors.

Gill and Sommerville explored whether 14-to 27-month-old toddlers use prior behavior to form expectations about future behavior within the moral domain, focusing on the sub-domains of fairness and help/harm. Their results suggest that infants utilize prior information from one moral sub-domain to form expectations of how an individual will behave in another sub-domain, especially after seeing hindering and unfair distributions. These findings provide evidence for a well-organized work of the moral domain.

On the development of prosociality, the next empirical contributions emphasizes that, in early childhood, helping, comforting and sharing behaviors are influenced by goal pursuit motivations, membership, and the social interaction contexts.

Karasewich et al. to examine the contexts in which shy children may be more or less likely to engage in prosocial behaviors, assessed 3.5- to 4.5-year-olds with prosocial problems, which were different from the type of intervention required (helping vs. comforting) and the source of the problem (social or object). Most of the children acted prosocially in the two helping tasks and in the object-centered comforting task, whereas shyer children were not less likely to intervene in all tasks. These findings provide insights into the methodological challenges of disentangling children's prosocial motivation, providing applicative implications.

Park and Jin investigated whether ingroup belonging reduces ingroup favoritism in 6-year-olds in terms of costly sharing, by applying a minimal-group paradigm. They found that children in the ingroup-exclusion and no-interaction conditions shared more resources with their ingroup members than outgroup members, while children in the ingroup-inclusion condition shared equally with the ingroup and outgroup members. These findings shed light on the role of the membership in sharing behaviors.

Hallers-Haalboom et al. examined the willingness of children to share more food with friends or acquaintances, through a previous published paradigm to replicate and extend knowledge on the topic (Birch and Billman, 1986). 3- to 6-year-olds were coupled with a friend or an acquaintance in a semi-natural context to assess the effect of food preference on sharing food and the interaction between type of relationship and sex. Overall, children were more willing to share non-preferred food. Additionally, boys were more likely to share with friends and girls with acquaintances. Only a partial replication of the results was found. The study highlights a growing need for replications and evaluation of the effects of socio-contextual aspects in more natural contexts.

Moreover, the additional empirical study highlights an interesting association between emotional comprehension, prosociality and conflict resolution strategies in early childhood.

Cao et al. investigated on the relation between emotional comprehension and peer conflict resolution strategies, by assessing 3-to 6-year-olds with the Test of Emotional Comprehension, and their preschool teachers with a Conflict Resolution Strategy Questionnaire. Their findings demonstrated that children with a good emotional comprehension have better prosocial behaviors, which in turn can positively predict the overall conflict resolution strategies.

Finally, the following literature review contributions highlights the role of social and interactive experiences and environmental factors on socio-moral development during the first years of life.

Tarsha and Narvaez investigated the influence of early social experiences on child neurobiological and sociomoral outcomes, specifically, the oxytocinergic system and prosociality, respectively. This review suggests that evolved nest components influence oxytocinergic functioning in parents and children, and contribute to the foundations for prosociality.

Carpendale and Wallbridge adopted a process-relational perspective and drew on developmental systems theory in arguing that infants born with emerging abilities to act and react without knowing about prosociality or morality. In their opinion, prosociality and morality emerge at the level of interaction within a human developmental system. The paper encourages reflection and helps to reconsider different theoretical perspectives that the literature presents today, which are equally interesting and significant contribution to better understand the early prosocial development.

Lu explored the link between goal pursuit motivation and prosociality in a pandemic context. Polish participants were randomly assigned to a simulated cartoon of the parable of the Good Samaritan, in which the normative focus on prevention or promotion was manipulated. The results confirmed a certain favorable trend toward offering help in both regulatory focus conditions, demonstrating a dynamic association between goal pursuit motivation and prosocial behavior.

Chen et al. implementing a bibliometrics approach, examined the dynamics and progress of the research agenda on moral education research. Specifically, they assessed basic quantitative information from studies published from January 2000 to September 2022, such as highly authors, organizations and countries, top-cited articles and journals. Then, applying cluster analyses, the authors organized the results. According to the authors, any research field is a dynamic process and requires effort from researchers to evaluate its evolution.

Limone and Toto investigated the overall contributing factors of the moral sense emergence and development, by a systematic review (PRISMA model). 26 studies were finally selected for the systematic review. Their findings suggested that, on the one hand, the moral sense appears to be an innate ability and that, on the other, social interactions and environmental factors can influence its expression and development.

## Perspectives

First studies on moral sense in infancy showed that by the end of the first year, infants possess a range of basic moral skills (e.g., Kuhlmeier et al., 2003; Hamlin et al., 2007, 2010, 2011; Geraci and Surian, 2011; Hamlin and Wynn, 2011; Sloane et al., 2012; Hamlin, 2013a,b). Subsequent research demonstrated that infants have the essential cognitive skills to make intuitive moral judgments in different prosocial contexts (for a review Woo et al., 2022). Moreover, other works found that infants can attribute causality, know others' intentions, and identify membership (e.g., Baillargeon et al., 2015). We have assumed that these early-emerging abilities, as well as a rich and multisensory set of experiences, can be the source of the infant's knowledge of what to expect from others in various situations. However, in contrast to other researchers who thought that these social skills are developed in a vacuum and isolation from the infant's early ties (e.g., Hamlin, 2013a), some researchers assume that participation in the dyadic interaction reorganizes intrapsychic and relational processes (Govrin, 2014, 2019). These processes become the foundations of understanding and experiencing regularities of behavioral patterns in other dyadic relations. The role of dyad (i.e., the mother-baby dyad) on socio-moral development remains still unclear and little explored.

Perhaps, the main limitation emerged from this Research Topic is the lack of attention by researchers to the role of the dyad, and family dynamics, in early moral development, therefore the influence of the socioemotional environment on this domain remains substantially unexplored.

As future directions, this Research Topic aims to suggest an alternative way for research on moral development, by proposing a more interactionist methodological approach that takes into account different variables. There is a lack of infant research about the determinants of moral development, to the point that the knowledge of moral development remains contested between different theoretical approaches, excluding the possibility of a dialogue that can shed light on some unknown aspects. We believe that new infant research is needed to address the early relations between social context and moral development (Kelley and Power, 2013; Ziv and Sommerville, 2017; Dahl, 2018).

## Author contributions

AG: Conceptualization, Data curation, Methodology, Resources, Supervision, Writing—original draft, Writing—review and editing. LF: Methodology, Data curation, Supervision, Visualization, Writing—original draft, Writing—review and editing. AG: Methodology, Data curation, Supervision, Writing—original draft, Writing—review and editing. PR: Conceptualization, Data curation, Methodology, Supervision, Writing—original draft, Writing—review and editing.
